# The Accommodation of Excess Charge in Binary Particle Lattices: A Many-Body Electrostatic Study

**DOI:** 10.1021/acs.jpcb.5c04192

**Published:** 2025-09-10

**Authors:** Holly Avis, Eric B. Lindgren, Benjamin Stamm, Elena Besley, Anthony J. Stace

**Affiliations:** † School of Chemistry, 6123 University of Nottingham, University Park, Nottingham NG7 2RD, United Kingdom; ‡ Departamento de Físico-Química, Instituto de Química, 28110 Universidade Federal Fluminense, 24020-141 Niterói, Rio de Janeiro, Brazil; § Institute of Applied Analysis and Numerical Simulation, 9149 University of Stuttgart, Pfaffenwaldring 57, 70569 Stuttgart, Germany

## Abstract

Many binary particle lattices are fabricated from charged particles on the assumption that the resultant structure is overall charge neutral. Results presented here from calculations on nine separate particle lattice types show that when both Coulomb and many-body multipole electrostatic interactions are taken into account, a lattice can actually gain stability by accommodating a small excess charge, either positive or negative. This effect arises from an increase in stability due to charge-induced multipole interactions, which serve to counteract destabilizing interactions that arise from repulsive Coulomb forces. It is shown that most of the lattice types considered could accommodate over 20% excess charge before becoming completely destabilized.

## Graphical abstract

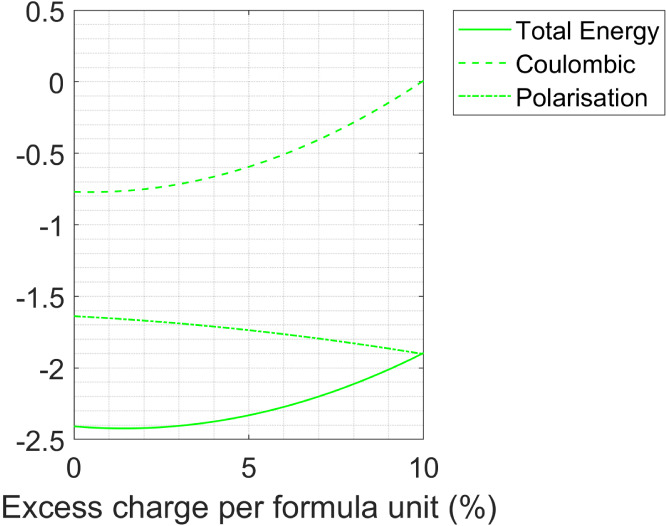

## Introduction

A number of experimental studies have examined how the presence of charge on nano- and microsized particles may facilitate the fabrication of binary lattices,^1–8^ which in many cases are sufficiently robust that they can be observed as stable solid structures.^6–8^ Although the formation, structure, and phase behavior of charged colloidal crystals in solution can be attenuated by changes to the Debye electrostatic screening length,^8^ once present as solid structures,^6–8^ stability depends primarily on the fundamental interactions arising from Coulomb and many-body forces together with van der Waals interactions. The development of solvent-free assembly methods, where charge is acquired through tribocharging or contact electrification, offers a route to self-assembly where the scale of electrostatic interactions can be characterized more easily.^9^ Contact electrification has been used extensively by Whitesides and coworkers to model the 2D assembly of oppositely charged millimeter-sized particles. This approach has been extended to three dimensions by Haeberle et al., but within the limitations imposed by a container;^10^ in contrast, Lee et al. have been able to observe three-dimensional assembly in the gas phase, but for small numbers of charged particles.^11^

How much excess or noncompensating charge, either negative or positive, a stable binary lattice could accommodate has received very little discussion. From quoted approximate values it is clear that there can be differences in charge between negative and positive particles;^1,2^ however, in two studies that have examined lattice structures where there were significant differences, the electrostatic interactions were substantially moderated by the presence of a solvent.^1,2^ It has been assumed that an extended three-dimensional lattice will only be stable if negative and positive particle charges compensate one another, and that repulsive Coulomb interactions will limit growth if there is an excess of either charge.^3,4^ The consequences of excess charge on the assembly of two-dimensional arrays of particles can be seen in the experiments of Whitesides and coworkers^12^ and the simulations of Lindgren et al.^13^ From these experiments it was concluded that the formation of stable, compact, close-packed assemblies required approximate electrical neutrality, and the presence of excess charge on one type of particle, led to the formation of chains and “rosettes”; the latter were small, mostly separate units consisting of a single particle of higher charge surrounded by oppositely, lower-charged particles.^12^ The formation of chains was also observed in the simulations and found to be as a consequence of the need to minimize repulsion between like-charged particles.^13^

Reported here are the results of calculations where a many-body theory of electrostatic forces has been applied to the investigation of interactions between collections of charged particles where the charges are not balanced; the intention being to examine the capacity for lattices to accommodate excess charge. Previously, the theory has been used to study a wide range of electrostatic interactions,^13–17^ and more recently to investigate the geometries of stable binary collections of charged, dielectric particles.^18^ In this latter study, it was possible to identify the contribution induced many-body electrostatic interactions made to lattice stability as a function of particle size ratio, γ. The calculations showed that for a number of examples, multipole interactions made a very significant (>80%) contribution to the lattice energy of arrays of negatively and positively charged particles. Those calculations have now been extended to explore the consequences of adding excess charge to stable, ordered binary arrays of polarizable dielectric particles. The stabilities of six stoichiometries, AB, AB_2_, AB_3_, AB_4_, AB_5_, and AB_6_, have been studied in the form of nine separate particle lattices that are isostructural with the following lattice types: NaCl, CsCl, CuAu, AlB_2_, MgZn_2_, AuCu_3_, CFe_4_, CaCu_5_, and CaB_6_.^3,4,19^ The unexpected result is that in the presence of a small amount (2%) of excess charge, either negative or positive, each of these structures *gains* stability and that with up to 20% excess charge a number of the lattice types show little evidence of complete instability. Both effects are driven by attractive polarization interactions, which are found to gain in strength as excess charge is added to a lattice, and these interactions serve to counteract increases in repulsive Coulomb interactions. One lattice type, CaB_6_, shows a positive Coulomb energy under all circumstances and is stable only because of the presence of attractive multipole interactions; however, such a structure is still able to accommodate excess charge. Estimates are given of the excess charge lattices could accommodate before they become completely unstable through dominant Coulomb interactions.

## Theory

A general solution based on an integral equation approach to the problem of calculating electrostatic interactions between large numbers of dielectric, spherical particles has previously been presented by Lindgren et al.^16^ Details regarding the application of the theory to binary lattices have been presented in a previous publication,^18^ where it has been shown that particle radius ratios that favor global minima in electrostatic energy are the same or a close match to those observed by experiment, and that many-body rather than two-body interactions are ultimately responsible for the observed structures.

In this work, the nine static structures with particle ratios identified as energy minima are examined with respect to the addition of excess charge, either negative or positive, to each particle. Each particle carries a free charge *q*_*i*_, uniformly distributed over the surface and is represented in the calculations by a surface density, $\sigma_{i}=q_{i}/\left(4\pi r_{i}^{2}\right)$ , where *r*_*i*_ is the radius. Coulomb and charge-induced multipolar interactions are calculated and the nonadditive nature of these interactions^14,15^ is taken into account through the mutual polarization of charged, dielectric particles. The model takes as input the number of particles, each with an assigned radius, dielectric constant (relative permittivity), charge, and position in three-dimensional space. Output consists of the distribution of surface charge, the electrostatic energy (Coulomb and induced), and the total force acting on each particle. The charge that is assigned, denoted here as *free* charge, is the quantity over which experimentalists generally have control; however, because the particles are considered to be composed of a dielectric material, they become polarized when in the presence of an external electric field, which in this context is generated by the presence of free charge on adjacent particles. Consequently, polarized bound charge accumulates on the surface of each particle, which leads to an anisotropic distribution of total (*free* + *bound*) surface charge.^18^ This is coupled with similar processes on all other particles via mutual polarization; a mechanism that can only be properly described within a many-body formalism.^16^ In the examples that follow, Coulomb and charge-induced multipolar interactions are considered up to the sixth degree (*N* = 6;64 pole). For calculations, involving large numbers of particles, the evaluation of multipole interactions benefits from the implementation of a fast multipole method (FMM),^16^ which provides a significant enhancement to the speed of computation, to the point where there is a linear scaling with respect to the number of particles and the time required for each computation of the interaction energy. The particles are assumed to be suspended in a homogeneous medium of dielectric constant *k*_0_, where in the present case *k*_0_ = 1 for air (approximately) or vacuum, which means that these calculations are more appropriate for crystals that have been deposited rather than held in solution.^9^ The consequence of including a solvent in a many-body treatment has recently been addressed,^20^ where it has been shown that the strength of the interaction between charged particles is strongly dependent on both the magnitude of the solvent’s dielectric constant and the ionic screening (Debye length) of the medium, while the sign of the interaction can be altered by the dielectric contrast between particles and medium.

Initial convergence tests were performed on lattices that were overall neutral to determine the most suitable size for each of the calculations.^16,18^ For each AB_*n*_ structure particle A was given a charge of *q*_1_ = +1 and each particle B a charge of *q*_2_ = −1/*n* to ensure neutrality.^16,17^ For all of the lattice types considered here, the total electrostatic energy per particle was found to have converged to within ≈0.4% once the lattice contained 1000 particles, and all subsequent calculations have used that number of particles to give an appropriate balance between computational cost and accuracy. Previous calculations showed that for the example of an atomic NaCl lattice, where anion and cation spheres appropriate for the sizes of Na^+^ and Cl^–^ were used, the lattice energy and the Madelung constant asymptotically approached their literature values as the number of charged particles increased.^16^

## Results and Discussion

For each of the stoichiometries listed above, the total electrostatic energy and the individual contributions of Coulomb and multipolar interaction energy have previously been calculated as a function of the particle radius ratio γ = *r*_small_/*r*_large_,^18^ where *r*_small_ is the radius of the smaller particle and *r*_large_ is the radius of the larger particle. For this study, a dielectric constant of 20 for both particles was used as a compromise between particles that are weakly polarizable, such as hydrocarbons and polymers, and particles that are strongly polarizable, for example, water droplets (ice), metal oxides, and metals. The consequences of changing the dielectric constant have been examined,^18^ and the results show that for certain lattice types where the many-body contribution was high, it remained so even for a dielectric constant as low as 2.^17^ In contrast, for particles in, for example, an NaCl lattice, where Coulomb interactions were found to dominate, that remained the case irrespective of the value assigned to the dielectric constant.^18^

The experimental literature on particle lattices covers charges ranging from ∼0 ± 1 to 10^6^ *e* ^3,4,11,12,17,21–24^ and particle sizes that range from nanometers up to micrometers,^3,4,12,21–24^ therefore, both particles were assigned arbitrary values that fell within these extremes. At the start of these calculations, each lattice type was overall neutral, and for a formula unit AB_*n*_ the charge on particle A, *q*_1_, was fixed at +1000 *e*, while the sum of the charges on each particle B was −1000 *e*, such that *q*_2_ = −1000/*n* *e*. Optimum values for γ, γ_ST_, corresponding to the most stable radius ratio, were determined by assigning each particle combination to an appropriate lattice, i.e., NaCl to a face-centered-cubic lattice, and then varying the radii of A and B particles within the range 50 to 500 μm while the particles remained in contact.^18^ These results are summarized in Table 1 and have previously been found to be a close match to most of the available experimental data.^3–5,18^

**Table 1:** Summary of Results Where Excess Charge Has Been Added to Individual Components of Each of the Listed Lattice Types^a^

Lattice type	γ_ST_ Calculated^b^	Particle with excess charge	*E*_min_/eV	Excess charge at *E*_min_/*e*	Gain in stability/eV^c^	Excess charge at *E* = 0 (%)^d^
NaCl	0.42	**Na**	–4.16	35	0.098	27
		Cl		18	0.029	25
CsCl	0.73	**Cs**	–2.41	20	0.027	20
		Cl		14	0.014	20
CuAu	0.91	**Cu**	–2.09	17	0.018	20
		Au		15	0.015	20
AlB_2_	0.58	Al	–1.81	20	0.023	20
		**B**		14	0.013	23
MgZn_2_	0.81	Mg	–1.81	19	0.022	20
		**Zn**		12	0.010	19
AlCu_3_	0.41	Au	–1.93	21	0.030	21
		**Cu**		14	0.017	20
CFe_4_	0.42^e^	**C**	–2.06	76	0.159	37
		Fe		6	0.014	28
CaCu_5_	0.46	Ca	–1.37	30	0.030	15
		**Cu**		9	0.003	20
CaB_6_	0.40	Ca	–0.92	23	0.031	24
		**B**		1	0.011	15

a *E*_min_ represents the new minimum energy per formula unit calculated with the excess charge shown. The smallest particle in each lattice type is shown in bold.

b Values of the particle radius ratio (γ) were calculated from minimum energy configurations where each lattice was overall neutral.

c Gain per formula unit.

d At this percentage excess charge on either A or B_*n*_ the total energy becomes zero and the lattice becomes unstable.

e Calculated for the ratio γ_ST_ (A/B) corresponding to *r*(C)/*r*(Fe).

For this study, the radius ratio corresponding to γ_ST_ for each lattice type, as given in Table 1, has been taken, and charge has been added stepwise to either particle A or particle B while optimizing the resultant charge distribution and each of the contributing energies, Coulomb and many-body. The results of these calculations are shown in Figures 1–3, where the Coulomb, many-body, and total energies have been plotted as a function of percentage excess charge. Since γ_ST_ equates to different values of *r*_small_ and *r*_large_, the consequences of adding excess charge to both components have been explored. A summary of the results is given in Table 1.

**Figure 1:**
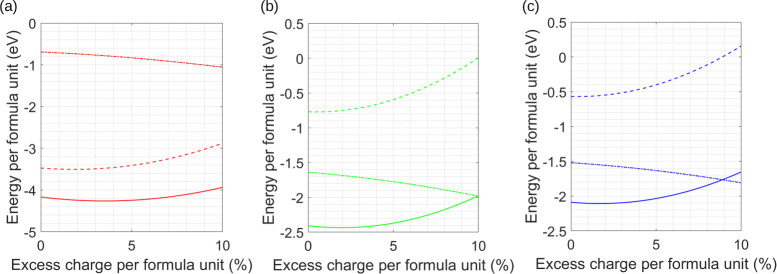
Changes in Coulomb (dashed line), induced many-body (dot-dashed line), and total energy (solid line) as a function of adding excess charge to the particle identified in an AB lattice. (a) Na in a NaCl lattice, (b) Cs in a CsCl lattice, and (c) Cu in a CuAu lattice.

**Figure 2:**
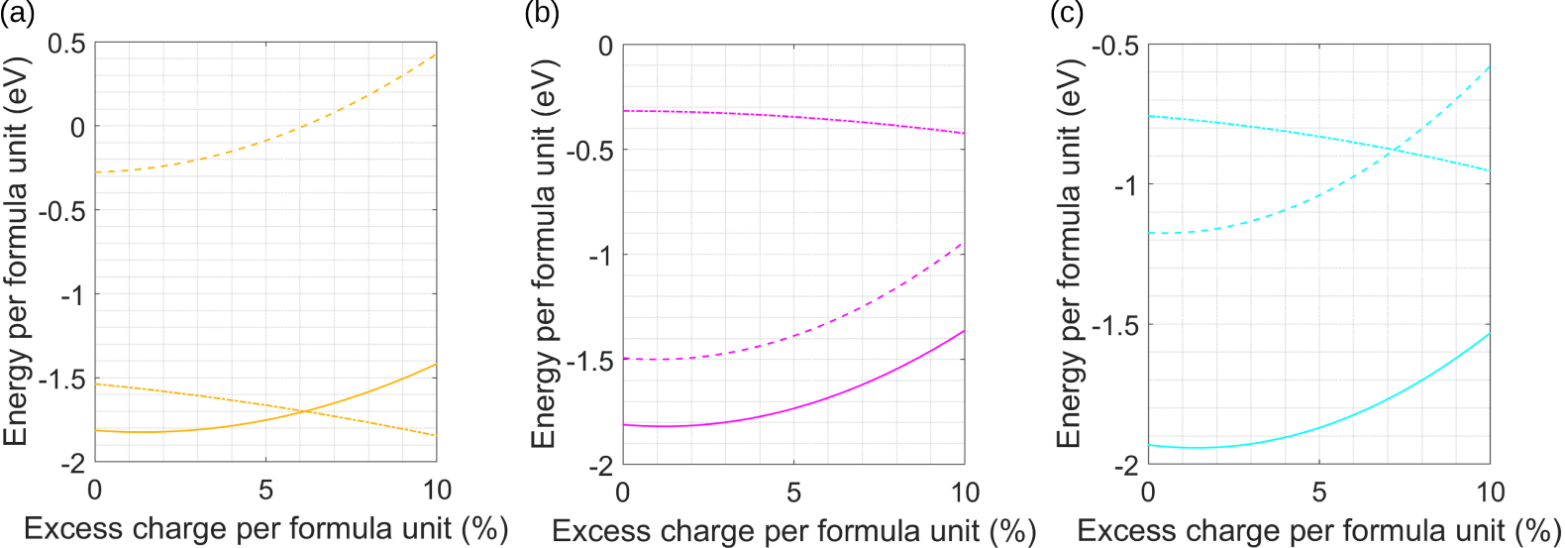
Changes in Coulomb (dashed line), induced many-body (dot-dashed line), and total energy (solid line) as a function of adding excess charge to the particle identified in either an AB_2_ or AB_3_ lattice. (a) B in an AlB_2_ lattice, (b) Zn in a ZnMg_2_ lattice, and (c) Cu in an AuCu_3_ lattice.

**Figure 3:**
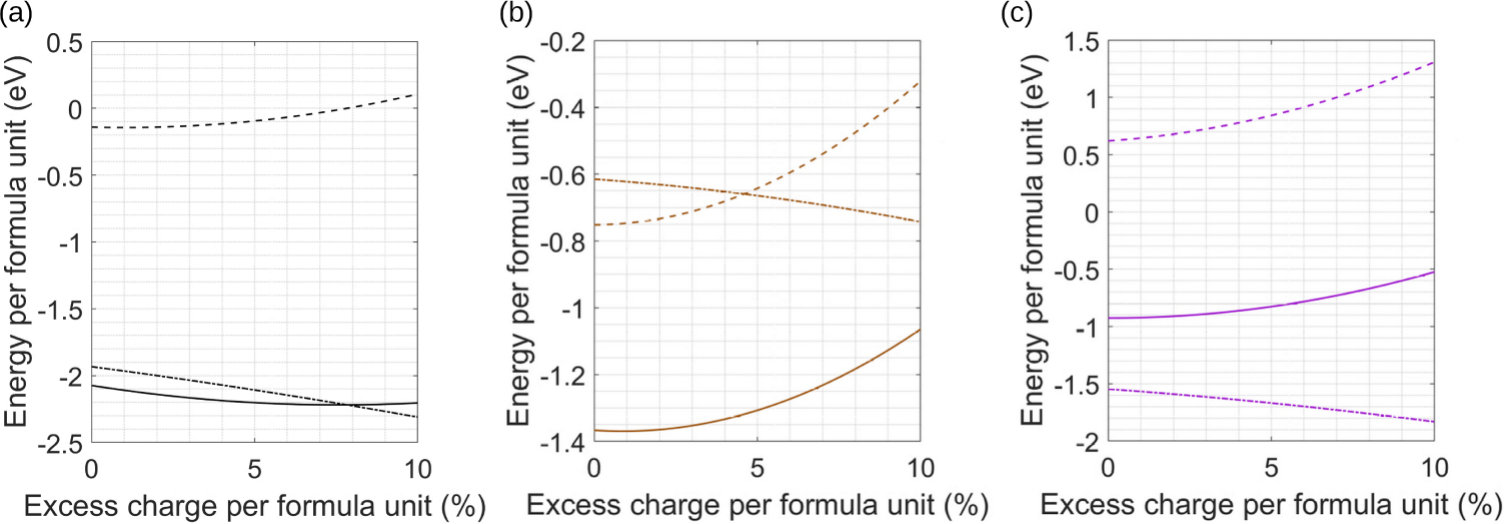
Changes in Coulomb (dashed line), induced many-body (dot-dashed line), and total energy (solid line) as a function of adding excess charge to the particle identified in either an AB_4_ lattice, an AB_5_ lattice, or an AB_6_ lattice. (a) C in a CFe_4_ lattice, (b) Cu in a CaCu_5_ lattice, and (c) B in a CaB_6_ lattice.

The results of calculations where excess charge has been added to each of the AB lattice types are shown in Figure 1a–c, where the individual electrostatic contributions to the total lattice energy have been plotted. Taking the NaCl lattice type as an example, Figure 1a shows results where excess positive charge has been added to the particle represented by Na in the lattice structure (starting at +1000 *e*), with the charge on the Cl particle remaining fixed at −1000 *e*; the value at which the lattice is initially overall neutral. In a complementary calculation, excess negative charge has been added to the particle represented by Cl with the charge on the Na particle remaining fixed at the neutral lattice value. However, the calculations showed that, for all AB_*n*_ lattice types, the graphs resulting from adding excess charge to either particle A or particle B have very similar profiles; therefore, for each example, just one set of results is given where excess charge on the particle identified has been gradually increased. At a qualitative level the patterns of behavior across all the figures are very similar; after a small lag, there is a sharp increase in the Coulomb energy, while the polarization energy becomes increasingly more attractive. There are, however, subtle differences between allocating charge to either A or B and these are summarized in Table 1. For all lattice types, the total energy, which is a balance between these two separate contributions, actually decreases following the addition of a small amount (∼2%) of excess charge (see Table 1), and as a consequence, each lattice type *becomes more stable with increased excess charge*.

As seen from Table 1, the gain in stability for the AB lattice types depends on whether charge is added to either A or B, with the larger increase coming from adding charge to the smaller of the particles, which results in a higher charge density, σ_*i*_. This observation is matched by the amount of excess charge that each particle can accommodate at *E*_min_. None of the three lattice types come close to being unstable with up to 10% excess charge, and the NaCl lattice is able to accommodate ∼25% excess before the total energy rises above zero. For CsCl and CuAu lattice types, the Coulomb energy becomes positive at ∼10% excess charge, but the many-body contributions maintain stability.

Figure 2a–c shows trends in the lattice stability as a function of excess charge for AB_2_ and AB_3_ lattice types. Referring to Table 1, it can be seen that it is the excess charge on A that results in the higher gain in stability; this again is a consequence of a higher charge density because any excess charge on B is now spread over two or three particles. The trends in energy are similar to those seen in Figure 1; however, for one example, the AlB_2_ lattice, the Coulomb energy again rapidly rises above zero, and it is only an enhanced contribution from the many-body interactions that maintain lattice stability. From Table 1 it can be seen that each lattice type is able to accommodate up to ∼20% excess charge before becoming unstable.

Figure 3a–c shows trends in lattice stability for three separate lattice types as a function of excess charge; these are CFe_4_, CaCu_5_, and CaB_6_. The patterns of behavior are similar to those seen in Figures 1 and 2; however, two of the examples warrant further discussion. First, the CFe_4_ lattice type is interesting because in this example, γ_ST_ corresponds to *r*(C)/*r*(Fe) and it is the central C that is the smaller of the particles. Hence, excess charge on this particle can generate a strongly polarizing interaction with the larger Fe particles, which, as seen in Table 1, results in comparatively large gains in both stability and the ability to accommodate charge. For the CaB_6_ lattice type, discussion of this structure’s stability has focused on entropy as the governing factor,^25^ and it had been noted previously that purely in terms of Coulomb interactions, this lattice should be unstable even as a structure where the charges balance to give overall neutrality.^18^ However, these latter calculations also identified the significant contribution many-body forces make to the stability of the lattice,^18^ and that is further confirmed by the results shown in Figure 3c. Overall, the calculations suggest that the CaB_6_ lattice should be able to accommodate at least 10% excess charge on the Ca particle without any significant loss of stability. Excess charge on the latter particle appears to enhance stability through polarization of the B particles; however, excess charge placed on B has a minimal effect; again, this is most probably because the charge is now spread over six particles.

All of the lattice types show a gain in stability with the addition of ∼2% excess charge to either of the particles, and although Table 1 shows these gains per formula unit to be comparatively small, if the assembled structures contain ∼1000 such units, then a complete lattice could gain an additional stability of ∼150 eV by accommodating just a few percent of excess charge. However, the effects seen in Figures 1–3 are dependent on the presence of initial charges, both positive and negative, on particles in a lattice that is overall neutral. If, for example, the initial charges on the particles in the NaCl-type lattice are reduced to ±100*e*, then the calculated trends in Coulomb and polarization energies are the same as seen in Figure 1a, but they and the total energy drop by 2 orders of magnitude. The requirement that the particles need to carry a charge in order to accommodate further excess charge is emphasized in Figure 4. Starting with a structure that has a CFe_4_ lattice type and where all of the particles involved are initially neutral, charge has been added to the particle in the C position. As can be seen, the behavior of the total energy as a function of charge follows the pattern expected of a multipole expansion and is dominated by Coulomb repulsion between the charged particles.^26^ Charge-induced multipole interactions between the neutral and charged particles are not sufficiently large to infer any stability of the lattice. Conversely, if the assembled particles carry a charge that is much larger than the 1000 *e* considered here (see, for example refs. 10–12,21–24 ), then the degree of stabilization and the ability to accommodate excess charge will be much more pronounced than what has been shown in these calculations.

**Figure 4:**
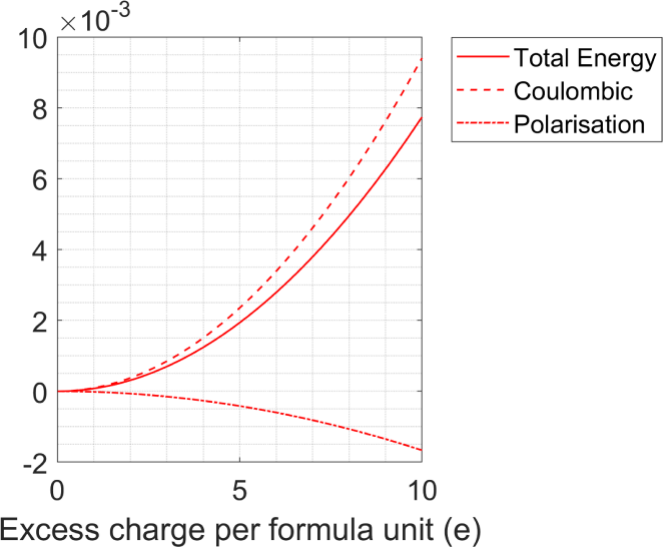
Changes in Coulomb (dashed line), induced many-body (dot-dashed line), and total energy (solid line) as a function of adding excess charge to particle C in a CFe_4_ lattice type. In this example, each particle in the initial lattice structure is neutral.

## Conclusion

Results from these new calculations on binary particle lattices show that when constructed from charged particles, an extended lattice could gain additional stability by accommodating a small excess charge, either positive or negative. For the case of a large lattice, the increased stability could be on the order of several hundred electron volts. This effect arises from a balance between repulsive Coulomb forces and attractive charge-induced multipole interactions, where the latter can effectively counteract the Coulomb forces with the addition of a few percent of excess charge. A possible outcome is that particle lattices, which were considered to be overall neutral, could retain an excess charge, either positive or negative, when prepared from charged particles.

## References

[1] Bartlett P., Campbell A. I. (2005). Three-dimensional binary superlattices of oppositely
charged colloids. Phys. Rev. Lett..

[2] Leunissen M. E., Christova C. G., Hynninen A.-P., Royall C. P., Campbell A. I., Imhof A., Dijkstra M., van Roij R., van Blaaderen A. (2005). Ionic colloidal
crystals of oppositely charged particles. Nature.

[3] Shevchenko E. V., Talapin D. V., Kotov N. A., O’Brien S., Murray C. B. (2006). Structural diversity in binary nanoparticle
superlattices. Nature.

[4] Shevchenko E. V., Talapin D. V., Murray C. B., O’Brien S. (2006). Structural
characterization of self-assembled multifunctional binary nanoparticle
superlattices. J. Am. Chem. Soc..

[5] Podsiadlo P., Krylova G. V., Demortière A., Shevchenko E. V. (2011). Multicomponent
periodic nanoparticle superlattices. J. Nanopart.
Res..

[6] Hueckel T., Hocky G. M., Palacci J., Sacanna S. (2020). Ionic solids from common
colloids. Nature.

[7] Zang S., Hauser A. W., Paul S., Hocky G. M., Sacanna S. (2024). Enabling three-dimensional
real-space analysis of ionic colloidal crystallization. Nat. Mater..

[8] Zang S., Paul S., Leung C. W., Chen M. S., Hueckel T., Hocky G. M., Sacanna S. (2025). Direct observation
and control of
non-classical crystallization pathways in binary colloidal systems. Nat. Commun..

[9] Jimidar I. S. M., de Waard M. T. J., Roozendaal G., Sotthewes K. (2024). Solvent-free confinement of ordered microparticle monolayers:
Effect of host substrate and pattern symmetry. Soft Matter.

[10] Haeberle J., Harju J., Sperl M., Born P. (2019). Granular ionic crystals
in a small nutshell. Soft Matter.

[11] Lee V., Waitukaitis S. R., Miskin M. Z., Jaeger H. M. (2015). Direct observation
of particle interactions and clustering in charged granular streams. Nat. Phys..

[12] Cademartiri R., Stan C. A., Tran V. M., Wu E., Friar L., Vulis D., Clark L. W., Tricard S., Whitesides G. M. (2012). A simple
two-dimensional model system to study electrostatic-self-assembly. Soft Matter.

[13] Lindgren E. B., Stamm B., Maday Y., Besley E., Stace A. J. (2018). Dynamic
simulations of many-body electrostatic self-assembly. Philos. Trans. R. Soc., A.

[14] Bichoutskaia E., Boatwright A. L., Khachatourian A., Stace A. J. (2010). Electrostatic analysis
of the interactions between charged particles of dielectric materials. J. Chem. Phys..

[15] Stace A. J., Boatwright A. L., Khachatourian A., Bichoutskaia E. (2011). Why like-charged
particles of dielectric materials can be attracted to one another. J. Colloid Interface Sci..

[16] Lindgren E. B., Stace A. J., Polack E., Maday Y., Stamm B., Besley E. (2018). An integral equation
approach to calculate electrostatic
interactions in many-body dielectric systems. J. Comput. Phys..

[17] Miller A., Halstead M., Besley E., Stace A. J. (2022). Designing
stable
binary endohedral fullerene lattices. Phys.
Chem. Chem. Phys..

[18] Lindgren E. B., Avis H., Miller A., Stamm B., Besley E., Stace A. J. (2024). The significance of multipole interactions for the
stability of regular structures composed from charged particles. J. Colloid Interface Sci..

[19] Bodnarchuk M. I., Kovalenko M. V., Heiss W., Talapin D. V. (2010). Energetic and entropic
contributions to self-assembly of binary nanocrystal superlattices:
Temperature as the structure-directing factor. J. Am. Chem. Soc..

[20] Lindgren E. B., Quan C., Stamm B. (2019). Theoretical analysis of screened
many-body electrostatic interactions between charged polarizable particles. J. Chem. Phys..

[21] Grzybowski B. A., Winkleman A., Wiles J. A., Brumer Y., Whitesides G. M. (2003). Electrostatic
self-assembly of macroscopic crystals using contact electrification. Nat. Mater..

[22] McCarty L. S., Winkleman A., Whitesides G. M. (2007). Electrostatic self-assembly of polystyrene
microspheres by using chemically directed contact electrification. Angew. Chem., Int. Ed..

[23] McCarty L. S., Whitesides G. M. (2008). Electrostatic charging due to separation of ions at
interfaces: Contact electrification of ionic electrets. Angew. Chem., Int. Ed..

[24] Soh S., Liu H., Cademartiri R., Yoon H. J., Whitesides G. M. (2014). Charging
of multiple interacting particles by contact electrification. J. Am. Chem. Soc..

[25] Ye X., Chen J., Murray C. B. (2011). Polymorphism in self-assembled AB6
binary nanocrystal superlattices. J. Am. Chem.
Soc..

[26] Stone, A. The Theory of Intermolecular Forces; 2nd ed.; Oxford University Press, 2013.

